# A High-Resolution Linkage Map Construction and QTL Analysis for Morphological Traits in Anthurium (*Anthurium andraeanum* Linden)

**DOI:** 10.3390/plants12244185

**Published:** 2023-12-17

**Authors:** Linbi Zhang, Yanyan Chen, Qingyun Leng, Xinge Lin, Jinping Lu, Yueting Xu, Haiyan Li, Shisong Xu, Shaohua Huang, Ariel López Hernán, Yaru Wang, Junmei Yin, Junhai Niu

**Affiliations:** 1 Tropical Crops Genetic Resources Institute, Chinese Academy of Tropical Agricultural Sciences (CATAS), Haikou 571101, Chinaxuyueting1325863@163.com (Y.X.); tarashiyumi@163.com (H.L.); wangyaru410@163.com (Y.W.);; 2 Institute of Crops Molecular Breeding, Henan Academy of Agricultural Sciences, Zhengzhou 450002, China; 3 The Engineering Technology Research Center of Tropical Ornamental Plant Germplasm Innovation and Utilization, Danzhou 571737, China; 4 Multidisciplinary Workshop on Vascular Plants, Border Ecology Laboratory, University of Flores, Sede Comahue (UFLO), Rio Negro 8328, Argentina; hernan_655@hotmail.com; 5 Botanical Garden of Plottier City, Neuquen 8316, Argentina

**Keywords:** anthurium, genetic linkage map, morphological traits, QTL analysis

## Abstract

*Anthurium andraeanum* Linden is a prominent ornamental plant belonging to the family Araceae and is cultivated worldwide. The morphology characteristics are crucial because they significantly impact ornamental values, commercial properties, and the efficiency of space utilization in production. However, only a few related investigations have been conducted in anthurium to date. In this study, an F_1_ genetic segregation population containing 160 progenies was generated through hybridization between potted and cut anthurium varieties. Fifteen morphological traits were assessed and revealed substantial levels of genetic variation and widespread positive correlation. Based on specific length amplified fragment (SLAF) sequencing technology, 8171 single nucleotide polymorphism (SNP) markers were developed, and the high-density linkage map of 2202.27 cM in length distributed on 15 linkage groups was constructed successfully, with an average distance of 0.30 cM. Using the inclusive composite interval mapping (ICIM) method, 59 QTLs related to 15 key morphological traits were successfully identified, which explained phenotypic variance (PVE) ranging from 6.21% to 17.74%. Thirty-three of those associated with 13 traits were designated as major QTLs with PVE > 10%. These findings offer valuable insights into the genetic basis of quantitative traits and are beneficial for molecular marker-assisted selection (MAS) in anthurium breeding.

## 1. Introduction

*Anthurium* Schott is the largest Neotropical genus of the family Araceae, composed of more than 950 genera and 2000 species, with a wide range of morphological diversity [1]. Anthurium is a flowering plant species, famous for its exotic shape, colorful spathe, and continuous blooming, that has been grown as a cut or potted flower worldwide [2,3]. To date, genetic research has been conducted mainly focused on the spathe color, blight resistance, and postharvest vase life [4,5,6,7]. Anthurium also shows a wide genetic diversity in morphological traits related to spathe, spadix, and leaf, which not only have a significant impact on its ornamental value but also determine the effective utilization of greenhouse space in production [8,9]. So, it is promising to develop novel varieties with distinct appearance structures to address market demands. However, due to the limited understanding of the genetic mechanisms underlying the complex morphology, breeding for morphological traits of anthurium has been time-consuming and costly.

In general, morphological traits are controlled by multiple genes, which can be identified through quantitative trait locus (QTL) mapping [10,11]. A series of genetic linkage maps were constructed and utilized for mapping various QTLs in ornamental plants, such as rose [12,13], carnation [14], chrysanthemum [15], lily [16,17], petunia [18], and tree pony [19]. Numerous linkage maps were constructed via traditional molecule markers such as RAPDs, SSRs, and AFLPs, which were limited by the number of reliable, repeatable, and stable markers [20,21,22,23]. In recent years, with the development of next-generation sequencing (NGS), simplified genome technologies, including the specific length amplified fragment sequencing (SLAF-seq), have been widely used for the development of single nucleotide polymorphism (SNP) markers and the construction of linkage maps [24]. To date, genetic linkage maps of many ornamental plants have been constructed using SLAF-seq technology, even polypoid plants with high heterozygosity and without reference genomes [25,26,27,28].

However, it is also challenging to construct a linkage map for anthurium due to the long generations, absence of pure lines, and complex heterozygosity generated by interspecific hybridization [29]. There is now only one genetic map available, which comprises 228 markers, including 99 RAPDs, 21 ISSRs, and 108 SRAPs, based on the interspecific F_1_ populations of 43 individuals descended from *A. ornatum* Schott and *A. andraeanum* Linden. The linkage map of *A. ornatum* was 1233.5 cM in length, distributed in 10 linkage groups (LGs), whereas the linkage map of *A. andraeanum* was 1023.5 cM in length, distributed in 12 LGs. The LGs were inconsistent with the haploid chromosome number of the *Anthurium* species (*n* = 15), and they only covered 77% and 73% of the genomes of *A. ornatum* and *A. andraeanum*, respectively [30]. Therefore, it is imperative to develop a genetic linkage map with a higher resolution to facilitate genetics and genomics research in anthurium.

In this study, SNP markers based on SLAF-seq technology were developed and the first high-density genetic linkage map of anthurium was constructed. Based on the map, QTLs related to 15 morphological traits were identified. It provides helpful genetics tools and information for molecular marker-assisted selection (MAS) in anthurium breeding.

## 2. Results

### 2.1. Phenotypic Analysis

**Figure 1:**
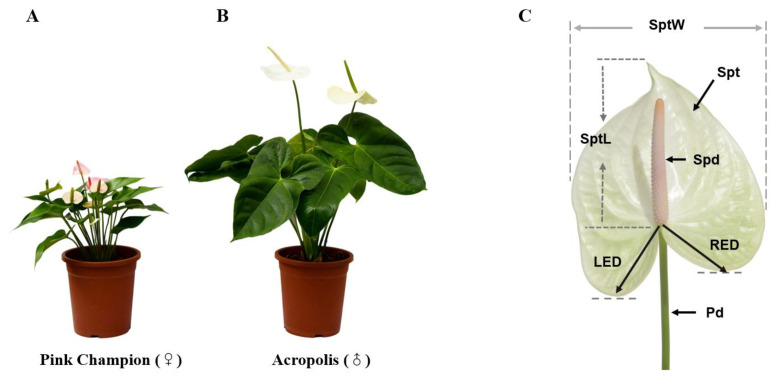
The phenotype of parents (**A**,**B**) and the schematic diagram of flower traits (**C**) in anthurium. Spt: spathe; SptW: spathe width; SptL: spathe length; LED: left ear distance; RED: right ear distance; Spd: spadix; Pd: pedicel.

Parameters of 15 morphological traits for the parents and F_1_ progenies were measured and statistically analyzed (Figure 1). The coefficients of variation (CV) of traits showed a high degree of genetic variation in the progenies (Table 1). The spathe left distance (LED) and right ear distance (RED), with the CV values of 34.04 and 35.53%, were significantly higher than other traits. Followed by spadix length (SpdL), spathe length (SptL), pedicel diameter (PedD), petiole length (PetL), spathe width (SpdW), and pedicel length (PedL), with values of 26.13%, 24.27%, 23.88%, 22.22%, 22.11%, and 21.82%, respectively. The CV of plant height (PH), leaf length (LL), leaf width (LW), and spadix diameter (top (SpdTD), middle (SpdMD), and base (SpdBD)) were all below 20%.

**Figure 2:**
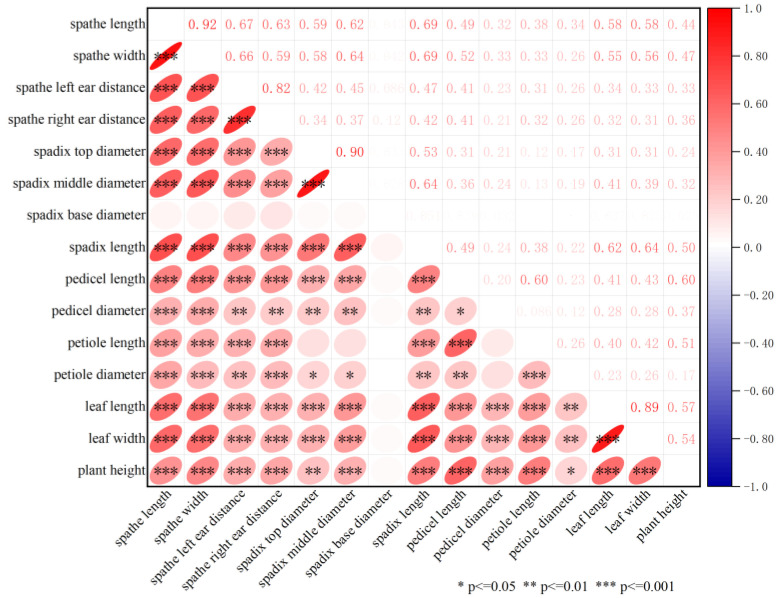
Pearson’s correlation coefficient and heatmap of 15 morphological traits in the F_1_ population.

Correlation analysis was conducted among 15 traits (Figure 2). The results indicated that the length, comprising spadix-, pedicel-, and petiole-, were significantly positively correlated with their diameter. The spathe length was strongly positively correlated with its width (r^2^ = 0.92, *p* < 0.001), the distance of the left and right ear in spathe was strongly positively correlated (r^2^ = 0.82, *p* < 0.001), and the leaf length was strongly positively correlated with its width (r^2^ = 0.89, *p* < 0.001). While the plant height was positively correlated with the length of pedicel and petiole.

**Figure 3:**
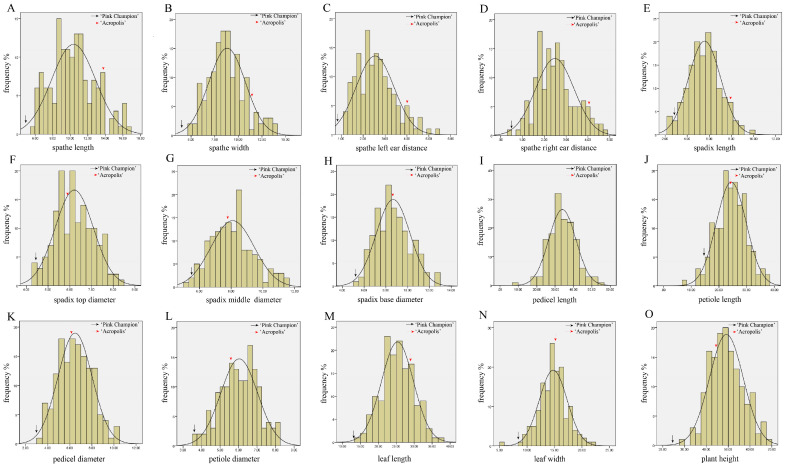
The distribution pattern for 15 traits measured in the mapping population. The subfigure (**A**–**O**) indicate the phenotypic variation of spathe-, spadix-, pedicel-, petiole-, leaf-, and height-related traits among the offspring, respectively. The normal distribution curve in the graph represented the expected percentage with respect to the measurement range of traits. The *x*-axis indicates the measurement values for each trait; the *y*-axis indicates frequency. The black and red arrows indicate the mean value of female and male parents, respectively.

The absolute values of skewness and kurtosis of 15 morphological traits in the 160 progenies were less than one with normal distributions. They were typical quantitative traits, which were suitable for QTL analysis (Figure 3).

### 2.2. SLAF Sequencing Data Analysis and Genotyping

**Figure 4:**
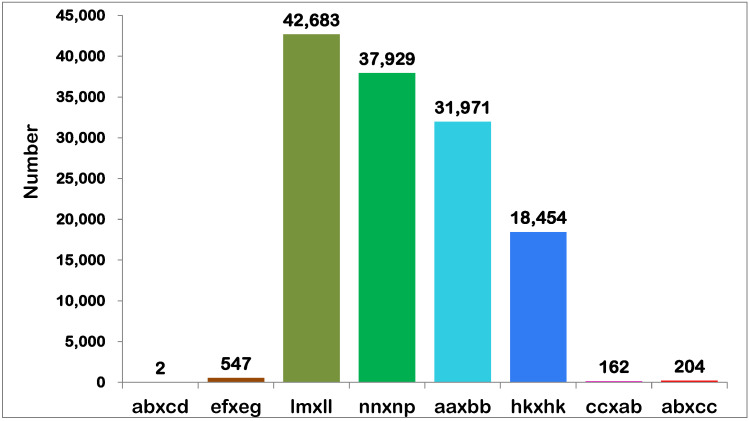
The markers distribution map of eight separation patterns in F_1_ progeny. The *x*-axis stated eight patterns of polymorphic SNPs markers; the *y*-axis stated the number of markers.

To genotype ‘Pink Champion’ (♀), ‘Acropolis’ (♂), and F_1_ progenies, SLAF-seq was performed and 188.37 Gb of raw data were obtained with a Q30 of 95.81% and GC content of 40.5%. The number of reads for the female and male parents was 9,869,344 and 11,383,208, and the mean for the F_1_ progeny was 5,790,723. The average sequencing depth was 80.65× for ‘Pink Champion’, 91.85× for ‘Acropolis’, and 31.68× for the F_1_ progeny (Table 2). Among these reads, 327,963 SNP markers were identified in all, of which 131,951 were successfully encoded and genotyped into eight segregation patterns (ab × cd, ef × eg, lm × ll, nn × np, aa × bb, hk × hk, cc × ab, ab × cc) (Figure 4). Filtered out 31,971 SNP markers were classified into the pattern of aa × bb (in the ratio of 24.23%), which is inapplicable to the CP model; the remaining 99,980 markers could be used for genetic map construction. To ensure a high-quality genetic map, low-quality SNP markers with integrity lower than 85%, parental information missing, and segregation separation *p* < 0.05 were removed. Finally, 10,648 SNP markers were identified for the genetic map construction.

### 2.3. High-Density Genetic Map Construction

**Figure 5:**
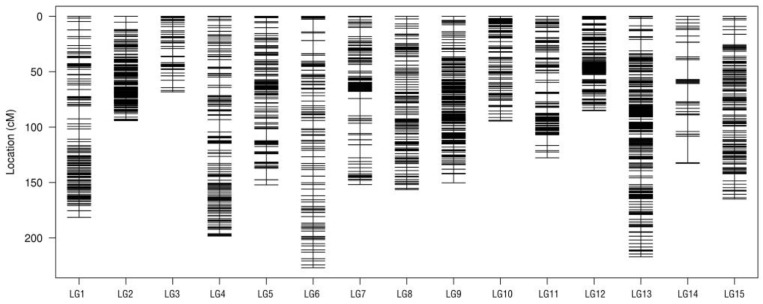
Genetic linkage map of the F_1_ population. The number at the bottom represents each linkage group. The ruler on the left indicates the genetic position in centimorgans (cM). The black bar represents the density of markers (cM/locus).

The modified logarithm of odds (MLOD) values were calculated between two SNP markers, and fewer than 10 were removed. A total of 8171 SNP markers (in the ratio of 76.48%) were ultimately retained for the high-density genetic map construction, which were distributed into 15 linkage groups (LGs). The total map distance of female and male parents was 2176.58 cM and 1940.36 cM, respectively. By integrating the parents’ genetic map, a linkage map with 2202.27 cM in length with an average distance of 0.30 cM was constructed (Figure 5 and Table 3). The largest linkage group was LG6 with a length of 226.98 cM which harbored 1082 markers, while the smallest linkage group was LG3 with 68.23 cM genetic distance containing 127 markers. The most saturated linkage group was LG12, which harbored 987 markers covering a length of 85.27 cM with the least average interval of 0.09 cM. The max gap on LG12 was only 4.46 cM, which is smaller than that of other linkage groups, while the largest genetic gap was found in LG14, with 23.88 cM genetic distance. The percentage of gap (<5 cM) was 98.40%, indicating that the markers were relatively well-distributed in the map [31]. According to a chi-square test (*p* < 0.05) of the 8171 SNP markers, 429 of which (with a ratio of 5.25%) were segregation distortion markers. The greatest number of segregation distortion markers were found in LG14, with a ratio of 33.83% (Table S1). 

Haplotype maps were constructed for each individual, and most recombination blocks were identified (Supplementary Figure S1). The average integrity of mapping markers was 99.72%, indicating the accuracy of genotyping and the high quality of the genetic map. Heatmap results indicated a strong linkage relationship between adjacent markers in the linkage group (Supplementary Figure S2). The haplotype map on the LG4 linkage group is shown in Supplementary Figure S3.

### 2.4. QTL Analysis of Morphological Traits

The inclusive composite interval mapping (ICIM) method was used to detect QTLs of morphological traits. A total of 59 associated significant QTLs were identified in two consecutive years, dispersed among 12 LG except for LG3, LG11, and LG14 (Table 4). Each QTL explained the phenotypic variance (PVE) ranging from 6.21% to 17.74%, while the LOD value ranged from 2.75 to 56.83. A total of 33 significant QTLs were detected and had the PVE above 10%.

For spathe traits, including spathe length (SptL), spathe width (SptW), left ear distance (LED), and right ear distance (RED), 18 QTLs were detected with the LOD values ranging from 3.60 to 56.83 and PVEs ranging from 6.21% to 17.74%, which were distributed on six LGs. For spathe length, four QTLs were detected on LG1 (*qSptL1*, 169.22 cM), LG6 (*qSptL2*, 7.34 cM), LG8 (*qSptL3*, 51.54 cM), and LG10 (*qSptL4*, 55.88 cM), in which two major QTLs were detected, and each explained 12.08% (*qSptL2*) and 12.67% (*qSptL3*) phenotypic variance. For spathe width, seven QTLs (*qSptW1*~*qSptW7*) were detected. In comparison, three major QTLs identified on LG6 (*qSptW2*, 7.34 cM), LG8 (*qSptW4*, 14 cM), and LG10 (*qSptW5*, 55.08 cM) explained 12.43%, 10.86%, and 12.71% phenotypic variance, respectively. For spathe left ear distance, a major QTL *qLED2* explained 17.21% of the phenotypic variance detected on LG12 (36.03 cM), and another QTL explained 9.51% of the phenotypic variance identified on LG1 (*qLED2*, 112.33 cM). For spathe right ear distance, five QTLs were detected, in which two major QTLs were detected on LG1 (126 cM, 130 cM), which explained 29.56% phenotypic variance. The QTL located on LG9 (25 cM) was consistently found in two environments with PVE of 12.91% and 8.06%, respectively.

For spadix traits, including spadix length (SptL), spadix top diameter (SpdTD), spadix middle diameter (SpdMD), and spadix base diameter (SpdBD), a total of 14 QTLs were detected with the LOD value ranging from 3.66 to 6.04, and each PVE ranged from 8.51% to 12.03%, which were distributed on five LGs. For spadix length, two major QTLs, *qSpdL1* and *qSpdL2*, were detected on LG2 (56.70 cM, 58.08 cM), which in total explained 23.95% phenotypic variance. For spadix top diameter, five QTLs were detected on LG1 (*qSpTD1*, 122.98 cM), LG6 (*qSpTD1*, 3.44 cM), LG8 (*qSpTD1*, 36.47 cM), and LG12 (*qSpTD1*, 75.26 cM; *qSpTD1*, 28.42 cM), with the LOD values ranging from 4.87 to 6.04 and each PVE ranged from 9.1% to 10.58%. For spadix middle diameter, a major QTL explained that 11% of the phenotypic variance was detected on LG1 (*qSpdMD1*, 125.56 cM). For spadix base diameter, two significant QTLs, *qSpdBD1* and *qSpdBD2,* were detected on LG1 (135.44 cM) and LG6 (6.07 cM), with each explaining 11.28% and 11.75% phenotypic variance, respectively.

For pedicel length (PdL), pedicel diameter (PdD), petiole length (PtL), petiole diameter (PtD), and plant height (PH) traits, 14 QTLs were detected with the LOD values ranging from 3.11 to 35.97, and each PVE ranging from 8.3% to 14.01%. For pedicel length, two major QTLs were detected on LG9 (*qPdL1*, 25.17 cM) and LG12 (*qPdL3*, 29.62 cM), which in total explained 22.64% phenotypic variance. While another two QTLs were detected on LG12 (*qPdL2*, 9.32 cM; *qPdL4*, 32.81 cM), explaining 8.81% and 8.78% phenotypic variance. For pedicel diameter, four QTLs were detected, while two major QTLs were identified on LG6 (*qPdD1*, 7.34 cM) and LG8 (*qPdD2*, 78.25 cM), explaining 11.16% and 10.85% phenotypic variance, respectively. Another two QTLs were detected on LG10 (*qPdD3*, 61.33 cM; *qPdD4*, 64.33 cM), which explained 21.65% phenotypic variance. For petiole length, two major QTLs were detected on LG4 (*qPtL1*, 26.29 cM) and LG7 (*qPtL2*, 5.82 cM), explaining 14.01% and 11.45% phenotypic variance, respectively. For petiole diameter, two QTLs were detected on LG12 (*qPtD1*, 29.55 cM) and LG15 (*qPtD1*, 12.91 cM) with PVEs of 9.94% and 9.83%, respectively. One QTL was detected on LG9 (127.45 cM) for plant height with PVE of 9.32%.

For leaf traits, including leaf length (LL) and width (LW), 13 QTLs were identified with the LOD value ranging from 3.14 to 6.69 and each PVE ranging from 8.47% to 13.23%, distributed on 8 LGs. For leaf length, six QTLs were found in the map, while four major QTLs were distributed on LG4 (*qLL2*, 44.40 cM), LG5 (*qLL4*, 100.48 cM), LG7 (*qLL5*, 60.07 cM), and LG9 (*qLL6*, 108.08 cM), which explained 13.23%, 10.78%, 11.59%, and 10.03% phenotypic variance, respectively. For leaf width, seven QTLs were identified, among which, four major QTLs were distributed on LG6 (*qLW2*, 7.34 cM), LG9 (*qLW3*, 108.06 cM), LG9 (*qLW4*, 119.05 cM), and LG12 (*qLW6*, 29.97 cM), which explained 11.49%, 12.77%, 11.77%, and 12.45%, respectively.

QTLs for spathe length co-localized with spathe width at 169.22 cM on LG1, 55.88 cM on LG10, and 7.34 cM on LG6; whereas QTLs for spadix length, spadix top, and middle diameter were co-localized at the position of 3.44 cM on LG6. The QTL for plant height was co-localized with leaf length at 127.45 cM on LG9. This suggested that some QTLs for morphological traits could be pleiotropy and/or exhibit a tight linkage.

## 3. Discussion

### 3.1. Constructing Linkage Map in Anthurium

Based on RAPD, ISSR, and SRKP molecular markers, one linkage map had been constructed in anthurium before. Due to the limited number of markers and small population size, the genetic map was not saturated enough, but a basic framework was formed [30]. In this study, a total of 327,963 SNP markers were identified by applying the SLAF-seq technique, of which 131,951 could be successfully encoded and genotyped. After a strict selection, 8171 high-quality SNP markers were used to construct a genetic linkage map, which is the highest number of markers and density to date, with an increase of 7934 markers compared to Venkat et al. [30].

Since ‘Pink Champion’ and ‘Acropolis’ were heterozygous clonal cultivars, clonal F_1_ progenies contain more alleles at each locus than bi-parental populations derived from two inbred parents, resulting in more significant genetic variation, it is difficult and specific to build a genetic map. By using the software HighMap, which is suitable for cross-pollinated (CP) populations [32], the genetic linkage map was constructed successfully with a total length of 2202.27 cM distributed in 15 linkage groups, which was consistent with the chromosomes in the *Anthurium* genus [33]. In particular, LG2, LG12, and LG15 were observed with the least inter marker distances, indicating maximum saturation and which might be considered to be recombination hotspots in this population. The mapping rate (mapped marker number/total marker number) was 76.48%. The average interval of 0.30 cM, and gap < 5 cM of 98.40%, indicate that the map was saturated and well-distributed with molecular makers, which were available for QTL analysis.

### 3.2. Segregation Distortion Markers

Segregation distortion is common in constructing a linkage map, where alleles in segregating populations deviate from the expected Mendelian ratio [34]. In employing this approach, segregation distortion leads to markers grouping errors and estimating recombination frequency in constructing a linkage map [35,36]. However, more studies have reported that only severe segregation distortion could affect mapping; therefore, containing distorted markers could improve the accuracy of marker grouping [37,38,39,40]. Therefore, we retained a considerable number of segregation distortion markers for constructing the linkage map in this study. A total of 8171 SNP markers were subjected to the chi-square test (*p* < 0.05) containing 429 high-quality segregation distortion markers (with a ratio of 5.25%, Table S1), which ensured the accuracy and genomic coverage of the linkage mapping.

### 3.3. QTLs Related to Morphological Traits 

The characteristics of spathe, spadix, pedicel, petiole, and leaf are essential components of commercial quality in anthurium. These components are therefore favorable targets for selection in breeding. Generally, the low and compact varieties with small bracts and short pedicels are used as potted flowers, while those with tall and loose plants, larger bracts, and longer pedicels are regarded as cut flowers. Although a few studies have been conducted on the morphology in anthurium [41,42], QTLs associated with morphological traits have not yet been reported. In this study, 15 main morphological traits in F_1_ population were measured for consecutive two years, showing a continuous distribution and variability and indicating their quantitative nature of inheritance, which were firstly mapped on the genetic linkage map in anthurium. A total of 59 significant QTLs with individual genetic effects were successfully detected, with each explaining phenotypic variance ranging from 6.21% to 17.74% and the logarithm of odds (LOD) values ranging from 2.75 to 56.83, of which 33 QTLs with PVE > 10% were associated with 13 traits and were designated as major QTLs. However, only one QTL (*qPH1*) related to plant height and two QTLs (*qPtD1*, *qPtD2*) associated with stem diameter with low PVE were identified, indicating that both are complex traits influenced by impacted small effect genes. Notably, most of the QTLs identified in this study are clustered in six locations in the genome (LG1, LG4, LG6, LG8, LG10, and LG12). These six regions contain multiple QTLs controlling different traits.

In addition, QTLs associated with different traits were observed co-localizing in the same interval on the same chromosome. QTLs for spathe length were co-localized with spathe width, pedicel diameter, and leaf width on LG1 (*qSptL1*, *qSptW1*), LG6 (*qSptL2*, *qSptW2*, *qPdD1*, *qLW2*), and LG10 (*qSptL4*, *qSptW5*). The QTL related to right ear distance (*qRED4*) was co-localized with pedicel length (*qPdL1*) on LG9 (25.17 cM). QTLs for right ear distance (*qRED2*), spadix middle (*qSpdMD2*), and base diameter (*qSpdBD1*) were co-localized on LG1 (130.34 cM). QTLs related to spadix length (*qSpdL3*) and spadix top diameter (*qSpdTD2*) were co-localized on LG6 (3.44 cM). The QTL for leaf length (*qLL6*) was co-localized with leaf width (*qLW5*) on LG9 (108.08 cM), while the QTL for plant height (*qPH1*) was co-localized with leaf width (*qLW3*) on LG9 (127.45 cM) (Table 4). The multiple-effect locus explained the prevalence of significant positive correlations among several different traits (Figure 2). The result that these QTLs associated with different traits were co-located might be attributed to one same QTL, a gene multi-effect, or two QTLs closely linked, therefore leading to the correlation among morphological traits. Similar co-localizing QTLs were also observed in other plants such as prunus mume [43,44], rice [45], wheat [46,47], sesame [48], cauliflower [49], barley, and so on [50]. However, whether the co-localized QTLs are single-gene with pleiotropism or are just closely linked but distinct genes in our results remains to be further studied.

Despite the huge potential of molecular markers in breeding programs, their implementation in MAS practice has been limited by the lack of information on the stability of QTLs across different environments and within different genetic backgrounds. Here, we present the results from the inheritance analysis of morphological traits within a population derived from a cross between the potted and cut anthurium cultivars over two successive seasons. However, further QTL analyses using multiple populations in larger size and different mapping methods would allow us to precisely position QTL mapping.

## 4. Materials and Methods

### 4.1. Plant Materials and DNA Extraction

The F_1_ population consisted of 160 progenies generated from the controlled cross between *Anthurium andraeanum* Linden cv. ‘Pink Champion’ (♀) and ‘Acropolis’ (♂). The two cultivars exhibit distinct field performances concerning morphological characters. ‘Pink Champion’ is a dwarf pot flower variety with a smaller blade and spathe, shorter pedicel, and spadix, while ‘Acropolis’ is relatively giant and widely used as a cut flower variety (Figure 1). Both F_1_ individuals and their parents were planted in the ornamental greenhouses of TCGRI-CATAS, Danzhou, China (109°42′ E/19°35′ N) and grown in a substrate consisting of 3: 1 (V:V) of composted coco blocks and coarse peat (pH 5.5–6.5) under the conditions of 18–30 °C and 60–80% relative humidity.

### 4.2. Phenotypic Measured and Statistical Analysis

Parameters of morphological traits for the parents and F_1_ progeny were measured in July 2017, April 2018, and December 2018. A total of 15 traits were measured, consisting of spathe length (SptL), spathe width (SptW), spathe left ear distance (LED), spathe right ear distance (RED), spadix length (SpdL), spadix top diameter (SpdTD), spadix middle diameter (SpdMD), spadix base diameter (SpdBD), pedicel length (PdL), pedicel diameter (PdD), petiole length (PtL), petiole diameter (PtD), plant height (PH), leaf length (LL), and leaf width (LW). The SpdTD, SpdMD, SpdBD, PdD, and PtD were measured with a vernier caliper; the other characteristics were measured using a ruler or tape ruler. The detailed measurement procedures were performed according to the study by Elibox [51].

For precise phenotyping, the measurements were conducted on the same growth stages for two consecutive years, and all traits were surveyed at least three times. The CV and MPs were analyzed with the software SPSS 26.0. The heatmap and correlation coefficient were created with the software OriginPro 2021 (v9.8.0.200).

### 4.3. SLAF library Construction and Sequencing

Healthy tender leaves (0.5–1.0 g) from the two parents and 160 F_1_ individuals were collected separately, and total DNA was isolated with the modified cetyl trimethylammonium bromide (CTAB) method [8]. DNA quality was visualized via electrophoresis in 1.0% agarose gels and the concentration was quantified using a DU800 Spectrophotometer (Beckman Coulter, Brea, CA, USA).

According to the estimated size of the anthurium genome (2.8 Gb) and GC content (40.5%), *Zea mays* were chosen as a reference genome to predict enzymatic digestion, deciding on a combination of endonucleases HinCII and SCal-HF. Subsequently, the digested fragment was subjected to add a 3′ end plus a nucleotide and then ligated to a dual-index sequence linker. Polymerase chain reaction (PCR) was performed using diluted restriction-ligation DNA samples and the primer pairs 5′-AATGATACGGCGACCACCGA-3′/5′-CAAGCAGAAGACGGCATACG-3′. PCR products were then purified using the QIAquick Gel Extraction Kit (Qiagen, Hilden, Germany). The target fragments with lengths of 314–414 bp were separated and sequenced on an Illumina HiSeq TM system. To check the accuracy of the library construction and sequencing, *Oryza sativa* subsp. *japonica* was selected as a control for the same protocol. To ensure the sequencing quality, the Q30 and GC content were calculated [52].

### 4.4. SNP Markers Detecting and Genotyping

SLAF marker identification and genotyping were performed following previous procedures [24]. The clean sequences from all samples were clustered based on sequence similarity. The SLAFs with two to four alleles were defined as potential polymorphic SLAFs, which can develop SNP markers and sort into eight segregation patterns (ab × cd, ef × eg, lm × ll, nn × np, aa × bb, hk × hk, cc × ab, and ab × cc). 

To ensure the quality of markers for the linkage map, the rules for SNP marker identification were as follows: (1) SNP markers with a complete degree > 85%. (2) SNP markers with significant segregation distortion (*p* < 0.05) were removed according to the chi-square test. (3) SLAFs with more than 5 SNP markers were filtered out, as it was considered a high-frequency variant region. (4) SLAFs with a sequencing depth of >25. (5) Remove redundant markers and the markers (aa × bb) that were not suited to the cross-pollination (CP) population type. 

### 4.5. Linkage Map Construction

The construction of a genetic map contains two essential components, grouping and ordering the markers. The MLOD values were calculated between two SNP markers to assign markers on the LGs, and the markers with MLOD < 10 were filtered out before ordering. The HighMap software with the CP option was used for anthurium genetic linkage map construction. The SMOOTH algorithm and the maximum likelihood method were used to correct genotyping errors and order the SNP markers in all LGs. The Kosambi algorithm was used to calculate genetic map distance (cM). Haplotype maps were drawn to detect double crossover populations and genotyping errors. Heatmaps were used to evaluate the relationship of recombination between markers from each LG.

### 4.6. QTL Analysis

The QTL analysis for the average of 15 morphological traits was performed via the ICIM method in GACD V1.0 [53]. The logarithm of odds (LOD) threshold for evaluating the statistical significance (*p* < 0.05) of each QTL was set by using a 1000 permutations test (PT), which is the LOD threshold for evaluating statistical significance. The potential locations of the QTLs were described according to their LOD peak locations and their surrounding regions. If no QTL was detected within the interval for some traits, the LOD score could be manually adjusted low, but it should not be lower than 2.0. A QTL was considered to be a major QTL if it accounted for more than 10% phenotypic variance.

## 5. Conclusions

In this study, a F_1_ segregation population with 160 individuals derived from the cross *Anthurium* cv. ‘Pink Champion’ (a potted variety) × ‘Acropolis’ (a cut variety) was used for constructing a high-density genetic linkage map. Based on SLAF-seq technology, 9134 SNP markers were developed and the first high-resolution genetic linkage map for anthurium was constructed. Fifty-nine QTLs associated with 15 main morphological traits were identified using the ICIM method. The results will lay a foundation for detecting genes related to morphological traits and MAS in anthurium breeding.

## Figures and Tables

**Table 1:** Descriptive statistics for morphological traits in anthurium parents and F_1_ population.

Traits	Parents		MPs	F_1_ Population					CV (%)
	‘Pink Champion’ X ± σ Mean ± SD	‘Acropolis’ X ± σ Mean ± SD		X ± σ Mean ± SD	Max	Min	Skewness	Kurtosis	
spathe length	4.76 ± 0.13	13.48 ± 0.27	9.12	10.37 ± 2.52	16.6	5.7	0.39	−0.28	24.27
spathe width	3.88 ± 0.14	11.64 ± 0.18	7.76	8.81 ± 1.95	14.0	4.9	0.52	0.03	22.11
spathe left ear distance	0.58 ± 0.07	3.98 ± 0.17	2.28	2.54 ± 0.87	5.4	1.2	0.70	−0.18	34.04
spathe right ear distance	0.42 ± 0.06	3.88 ± 0.33	2.15	2.48 ± 0.88	4.8	0.4	0.70	−0.03	35.53
spadix length	2.72 ± 0.10	8.00 ± 0.08	5.36	5.57 ± 1.46	10.1	2.4	0.18	−0.59	26.13
spadix top diameter	4.43 ± 0.17	5.69 ± 0.20	5.06	6.21 ± 0.88	8.28	4.32	0.10	−0.59	14.18
spadix middle diameter	5.46 ± 0.19	7.84 ± 0.22	6.65	8.09 ± 1.36	11.45	5.1	0.39	−0.18	16.80
spadix base diameter	5.77 ± 0.15	8.47 ± 0.14	7.12	8.67 ± 1.55	12.73	5.45	0.33	−0.38	17.93
pedicel length	19.58 ± 0.86	36.00 ± 1.00	27.79	33.94 ± 7.40	56.4	9.4	0.09	0.62	21.82
pedicel diameter	3.17 ± 0.08	6.25 ± 0.12	20.74	6.47 ± 1.55	10.23	3.15	0.21	−0.52	23.88
petiole length	15.04 ± 1.06	26.4 ± 1.87	20.74	24.44 ± 5.43	37.4	7.47	−0.13	0.03	22.22
petiole diameter	3.62 ± 0.19	5.53 ± 0.28	4.58	6.03 ± 0.99	8.1	3.71	−0.14	−0.47	16.49
leaf length	13.28 ± 0.69	28.66 ± 0.80	20.97	25.33 ± 4.49	37.2	14.5	0.11	−0.32	17.72
leaf width	6.96 ± 0.63	15.04 ± 0.54	11.00	14.76 ± 2.54	21.8	5.7	−0.10	0.83	17.20
plant height	24.42 ± 0.73	44.74 ± 1.22	34.58	49.15 ± 7.78	67.8	29.7	0.22	−0.36	15.82

MPs: mid-parent value; Max: maximum value; Min: minimum value; CV: coefficient of variation.

**Table 2:** Statistical sequencing data for the two parents and F_1_ progeny.

Sample	Total Reads	Total bases	Q30 Percentage (%)	GC Percentage (%)	Average Depth
‘Pink Champion’	9,869,344	1,973,515,426	96.38	39.40	80.65×
‘Acropolis’	11,383,208	2,276,329,712	96.53	39.29	91.85×
F_1_ progeny	5,790,723	1,157,976,894	95.80	40.53	31.68×
*Oryza sativa* subsp. *japonica*	404,628	80,919,752	95.32	37.6	
Total	941,977,531	188,368,171,360	95.81	40.5	

**Table 3:** Marker information for the high-density genetic map.

Linkage Group ID	Total Distance (cM)	Total Marker	Average Distance (cM)	Gap < 5 cM (%)	Max Gap (cM)
LG1	181.48	290	0.63	97.23	11.63
LG2	94.19	509	0.19	99.61	6.68
LG3	68.23	127	0.54	97.62	6.77
LG4	198.39	230	0.87	97.38	11.16
LG5	152.14	266	0.57	98.49	10.71
LG6	226.98	1082	0.21	98.89	11.97
LG7	151.14	573	0.27	98.95	15.63
LG8	156.36	678	0.23	99.85	5.34
LG9	150.31	615	0.24	99.67	8.1
LG10	94.5	211	0.45	100.00	4.58
LG11	127.73	338	0.38	97.63	9.57
LG12	85.27	987	0.09	100.00	4.46
LG13	217.11	973	0.22	99.59	10.38
LG14	132.74	1.33	1.00	91.26	23.88
LG15	164.93	1159	0.14	99.74	9.37
Total	2202.27	9341	0.24	98.40	23.88

**Table 4:** List of major QTLs identified by inclusive composite interval mapping.

Phenotypic Traits	QTLs	LGs	Position (cM)	Left Marker	Right Marker	LOD Threshold	LOD	PVE(%)
Spathe length	*qSptL1* ^a^	1	169.22	Marker125870	Marker86061	4.00	4.52	6.21
	*qSptL2* ^b^	6	7.34	Marker29182	Marker27771		7.61	12.08
	*qSptL3*	8	51.54	Marker22818	Marker25881		8.18	12.67
	*qSptL4* ^c^	10	55.88	Marker75159	Marker9344		56.83	7.59
Spathe width	*qSptW1* ^a^	1	169.22	Marker125870	Marker86061	3.50	3.82	9.96
	*qSptW2* ^b^	6	7.34	Marker29182	Marker27771		8.94	12.43
	*qSptW3*	6	16.06	Marker129013	Marker17981		3.9	8.84
	*qSptW4*	8	14.45	Marker15415	Marker18261		5.28	10.86
	*qSptW5* ^c^	10	55.08	Marker75159	Marker9344		5.35	12.71
	*qSptW6*	10	63.80	Marker124314	Marker10075		6.55	8.31
	*qSptW7*	12	17.15	Marker1346	Marker4679		4.07	8.36
Spathe left ear distance	*qLED1*	1	112.33	Marker7131	Marker130377	3.50	3.93	9.51
	*qLED2*	12	36.03	Marker9051	Marker3948		6.76	17.21
Spathe right ear distance	*qRED1*	1	126.35	Marker25500	Marker1420	3.60	32.43	17.74
	*qRED2* ^e^	1	130.34	Marker17440	Marker21610		25.26	11.82
	*qRED3*	8	22.25	Marker131050	Marker15414		3.6	12.79
	*qRED4* ^d^	9	25.17	Marker12185	Marker30201		4.71	12.91
Spadix length	*qSpdL1*	2	56.70	Marker46740	Marker63007	3.60	3.66	12.03
	*qSpdL2*	2	58.08	Marker63007	Marker38589		3.78	11.92
	*qSpdL3* ^f^	6	3.44	Marker68802	Marker28040		4.36	9.74
	*qSpdL4*	6	12.24	Marker36909	Marker129577		3.74	9.38
Spadix top diameter	*qSpdTD1*	1	122.98	Marker5630	Marker124889	3.00	5.81	10.58
	*qSpdTD2* ^f^	6	3.44	Marker68802	Marker28040		6.04	9.1
	*qSpdTD3*	8	36.47	Marker21492	Marker98352		4.87	10.47
	*qSpdTD4*	12	75.26	Marker29474	Marker10355		5.46	9.62
	*qSpdTD5*	12	28.42	Marker129430	Marker5235		5.97	10.37
Spadix middle diameter	*qSpdMD1*	1	125.56	Marker124889	Marker25500	2.50	4.08	11
	*qSpdMD2* ^e^	1	130.34	Marker17440	Marker21610		4.61	9.47
	*qSpdMD3*	6	2.44	Marker8889	Marker28041		2.75	8.51
Spadix base diameter	*qSpdBD1* ^e^	1	130.44	Marker17440	Marker21610	2.50	4.09	11.28
	*qSpdBD2*	6	6.07	Marker124707	Marker32959		5.3	11.75
Pedicel length	*qPdL1* ^d^	9	25.17	Marker12185	Marker30201	3.50	3.52	11.84
	*qPdL2*	12	99.32	Marker27459	Marker28180		4.12	8.81
	*qPdL3*	12	29.62	Marker44684	Marker44683		8.31	13.85
	*qPdL4*	12	32.81	Marker18483	Marker125128		5.85	8.79
Pedicel diameter	*qPdD1* ^b^	6	7.34	Marker29182	Marker27771	3.50	4.03	11.16
	*qPdD2*	8	78.25	Marker8125	Marker49700		3.71	10.85
	*qPdD3*	10	61.33	Marker9341	Marker124314		26.96	8.3
	*qPdD4*	10	64.33	Marker10075	Marker7177		35.97	13.35
Petiole length	*qPtL1*	4	26.29	Marker127434	Marker125835	4.10	4.17	14.01
	*qPtL2*	7	5.82	Marker27651	Marker48346		7.98	11.45
	*qPtL3*	12	64.64	Marker1990	Marker56730		6.58	9.06
Petiole diameter	*qPtD1*	12	29.56	Marker5470	Marker22855	3.20	3.3	9.94
	*qPtD2*	15	12.91	Marker26166	Marker126412		3.23	9.83
Plant height	*qPH1* ^h^	9	127.45	Marker12865	Marker37220	3.00	3.11	9.32
Leaf length	*qLL1*	1	110.17	Marker77752	Marker130680	3.00	4.2	8.96
	*qLL2*	4	44.40	Marker22748	Marker7385		4.54	13.23
	*qLL3*	4	99.04	Marker20320	Marker26418		4.64	8.47
	*qLL4*	5	100.48	Marker129900	Marker129902		4.23	10.78
	*qLL5*	7	60.07	Marker124796	Marker66308		4.4	11.59
	*qLL6* ^g^	9	108.08	Marker122327	Marker7345		3.14	10.03
Leaf width	*qLW1*	2	59.54	Marker63007	Marker38589	3.50	4.92	9.18
	*qLW2* ^b^	6	7.34	Marker29182	Marker27771		4.42	11.49
	*qLW3* ^h^	9	108.06	Marker122327	Marker7345		4.9	12.77
	*qLW4*	9	119.05	Marker78056	Marker18037		6.69	11.77
	*qLW5* ^g^	9	127.45	Marker12865	Marker37220		4.77	8.55
	*qLW6*	12	29.97	Marker44683	Marker33277		4.75	12.45
	*qLW7*	12	36.91	Marker124398	Marker27267		3.57	8.68

LGs: linkage groups. PVE: the phenotypic variation explained. LOD: the logarithm of odds. The LOD threshold for evaluating the statistical significance (*p* < 0.05) of each QTL was set by using a 1000 permutations test. The QTLs are labeled with the same letter (a~h) on the upper right and the same background color, indicating the co-localized loci for different traits.

## Data Availability

Original data is available upon request from the corresponding author.

## Supplementary Materials

The following supporting information can be downloaded at: https://www.mdpi.com/article/10.3390/plants12244185/s1, Figure S1. The integrity distribution map of all. Figure S2. the heatmap of LG4. Figure S3. The haplotype map of LG4. Table S1: The markers showing segregation distortion in mapping population.
