# Supplementary material for: A High-Resolution Linkage Map Construction and QTL Analysis for Morphological Traits in Anthurium (Anthurium andraeanum Linden)
Source: Plants (Basel). 2023 Dec 17;12(24):4185. doi: 10.3390/plants12244185 (PMC10747322; doi:10.3390/plants12244185)
Supplement: Supplementary file 1 [file plants-12-04185-s001.zip › plants-2703352-supplementary Table S1.pdf]

Table S1. The markers showing segregation distortion in mapping population.

Linkage Group ID	SLAF marker number	Segregation distortion marker number	Segregation distortion marker percentage（%）
LG1	290	23	7.93
LG2	509	21	4.13
LG3	127	1	0.78
LG4	230	15	6.52
LG5	266	74	27.82
LG6	1,082	74	6.84
LG7	573	32	5.59
LG8	678	45	6.64
LG9	615	25	4.07
LG10	211	7	3.12
LG11	338	11	3.25
LG12	987	13	1.34
LG13	973	26	2.67
LG14	133	45	33.83
LG15	1,159	17	1.47
Total	8,171	429	5.25
