# Supplementary material for: A High-Resolution Linkage Map Construction and QTL Analysis for Morphological Traits in Anthurium (Anthurium andraeanum Linden)
Source: Plants (Basel). 2023 Dec 17;12(24):4185. doi: 10.3390/plants12244185 (PMC10747322; doi:10.3390/plants12244185)
Supplement: Supplementary file 1 [file plants-12-04185-s001.zip › Supplementary Figure legends.pdf]

Supplementary Figure legends

Figure S1. The integrity distribution map of all individuals. The x-axis represents the 160 individuals and y-axis represents the complete degree of mapped markers.

Figure S2. **The heat maps for LG4.** Each row and column are markers arranged in the order of the graph. Each small square represents the recombination rate between two markers, and the color change from yellow to red to purple represents the recombination rate change from small to large. Markers with proximity have a low recombination rate and a color close to yellow. Markers that are far away have a high recombination rate, approaching purple.

Figure S3. **The haplotype map for LG4. Each** horizontal row represents a marker, each column represents an individual sample. Green represents the first allele from the parent, blue represents the second allele from the parent, white represents undetermined, and gray represents missing. The location where the same column color changes is the location where the recombination event occurs.
