# Supplementary figures and images for: A High-Resolution Linkage Map Construction and QTL Analysis for Morphological Traits in Anthurium (Anthurium andraeanum Linden)
Source: Plants (Basel). 2023 Dec 17;12(24):4185. doi: 10.3390/plants12244185 (PMC10747322; doi:10.3390/plants12244185)

# Individuals Integrity

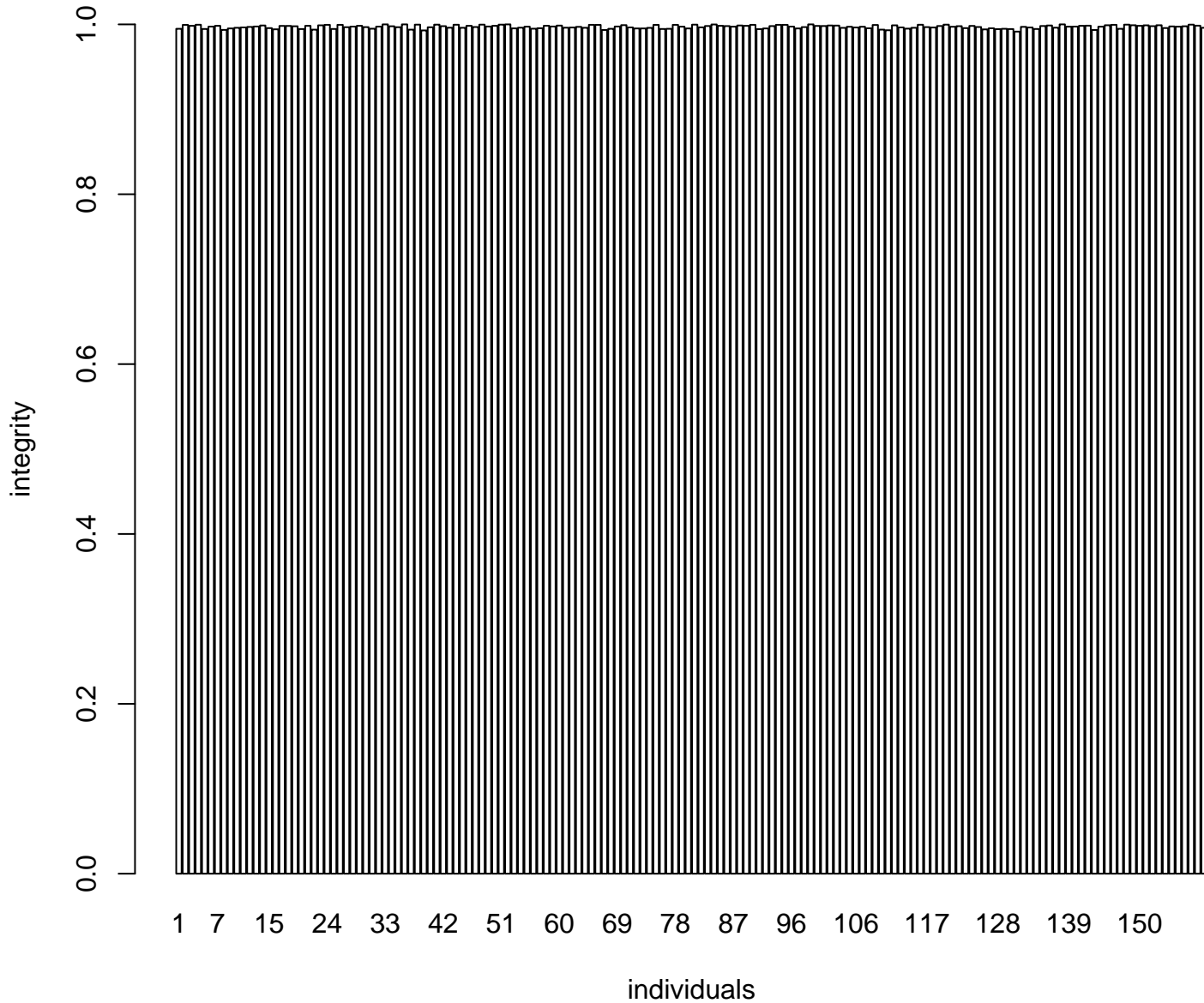

Supplement: Supplementary file 1 [file plants-12-04185-s001.zip › Figure S1. The integrity distribution map of all individuals..pdf]

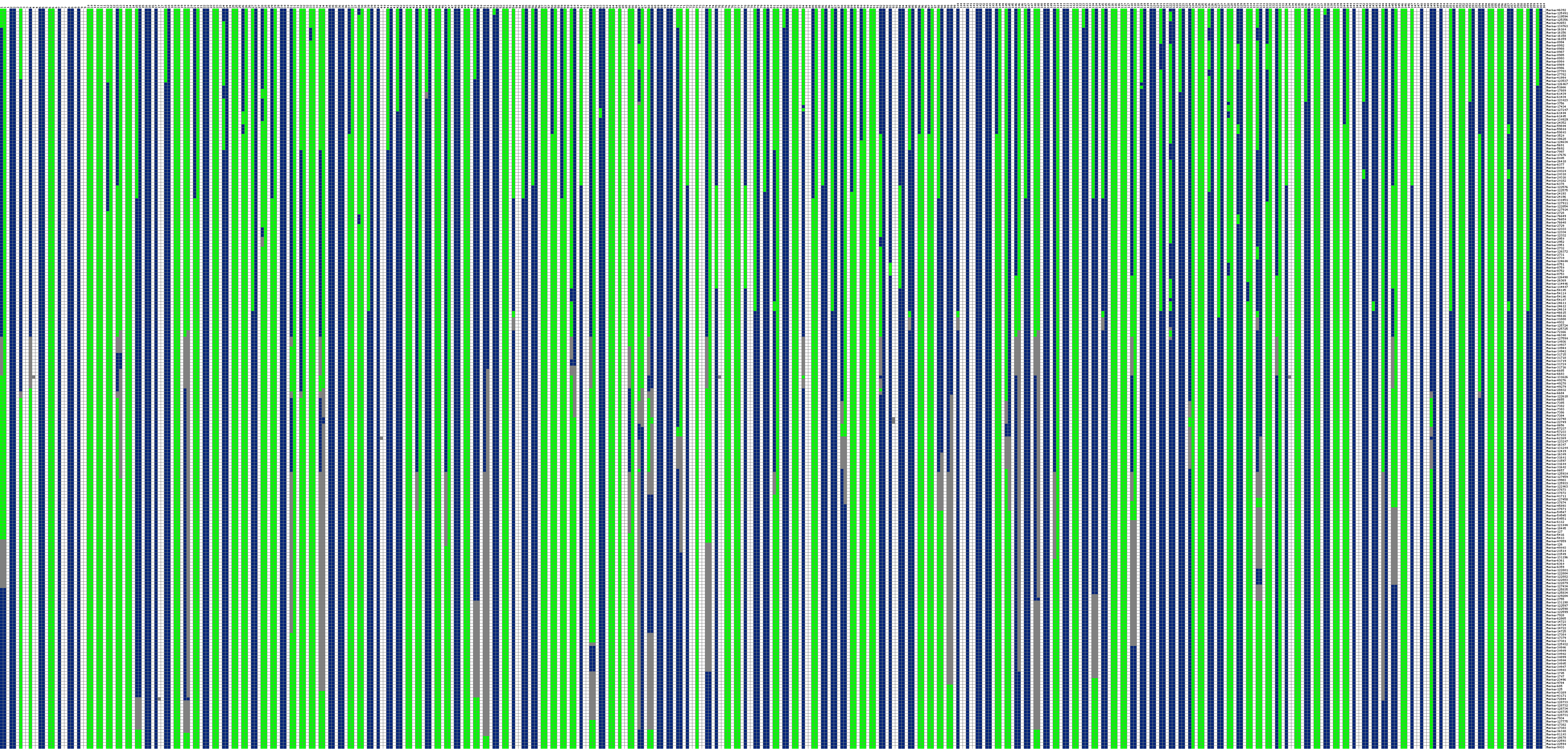

Supplement: Supplementary file 1 [file plants-12-04185-s001.zip › Figure S2. The heat map for LG4..png]

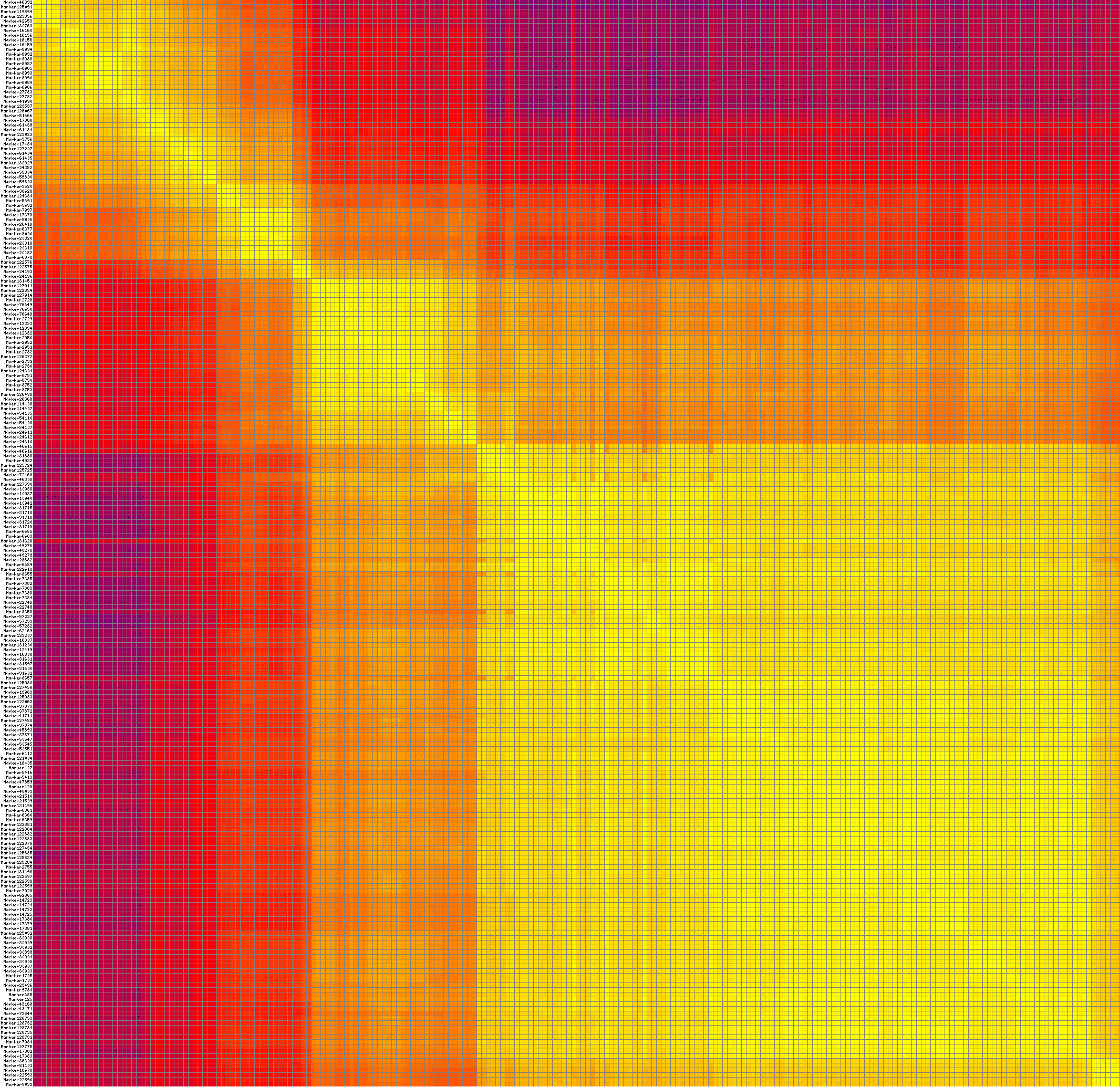

Supplement: Supplementary file 1 [file plants-12-04185-s001.zip › Figure S3. The haplotype map for LG4..png]
